# Supplementary material for: DNA Methylation in Offspring Conceived after Assisted Reproductive Techniques: A Systematic Review and Meta-Analysis
Source: J Clin Med. 2022 Aug 28;11(17):5056. doi: 10.3390/jcm11175056 (PMC9457481; doi:10.3390/jcm11175056)
Supplement: Supplementary file 1 [file jcm-11-05056-s001.zip › jcm-1846663-supplementary.pdf]

Supplementary Table S1. Findings of the included studies

Author and year	Findings	Limits
Argyraki et al., 2022	• No difference in the methylation and expression levels of the 3 imprinted genes analyzed	• Small sample size;<br>• No info on paternal infertility<br>• The difference between gender was not analyzed
Barbaret et al., 2021a	• Hypomethylation of *H19/IGF2* DMR in ART group than in controls confirmed with pyrosequencing<br>• Hypermethylation of *PEG3* DMR in ART group than in controls confirmed with pyrosequencing<br>• In children born by ICSI presence of hypomethylation of *LINE1* compared with IVF<br>• Identification of DMRs located in the promoter region of the *MEG3*, *BLCAP*, and *DLX5* genes by EPIC array<br>• No differences in the ART group between conception method (IVF and ICSI) and culture medium used	• Small sample size<br>• No info on parental infertility<br>• Possible distant epigenetic effects considering they are 7-8 years old children<br>• Single or twin pregnancies were not specified<br>• The difference between gender was not analyzed
Barberet et al., 2021b	• Hypomethylation of *H19/IGF2* DMR in the fresh ET group than in the frozen ET and compared with the placenta-level control even after adjustment for maternal age, parity, sex of the unborn child, and term of pregnancies<br>• Hypomethylation of *LINE1* and HERV-FRD in the fresh-ET vs frozen-ET group and toward the controls even after adjustment for maternal age, parity, sex of the unborn child, and term of pregnancies<br>• Decreased expression of *LINE1* ORF2 in the placentas and cord blood of babies born from IVF/ICSI and fresh ET<br>• Reduced expression of *H19* in frozen than in fresh ET<br>• Methylation levels of *H19/IGF2* and HERV-FRD and the expression of *KCNQ1* were lower in the placentas from IVF than those from ICSI	• Small sample size<br>• No info on paternal infertility<br>• Only a few genes analyzed
Camprubì et al., 2013	• The methylation of DMRs was comparable in the placenta among groups<br>• Pyrosequencing did not detect any differences between the groups even at the level of *Alu Yb8*, *LINE1*, α-satellite repeats, and in the promoters of the genes analyzed	• Heterogeneous population<br>• Analysis of subgroups performed on small numbers<br>• No information on paternal infertility<br>• No information on the type of ART method used

		• The difference between gender was not analyzed
Caramaschi et al., 2011	• After adjustment for various maternal risk factors such as age, BMI, and smoking, observed 5 CpGs hypermethylated of which 2 were located in the *ARRDC4* gene and 1 in the *SRD5A2* gene<br>• Association of these methylation alterations with COS	• No info on paternal factor<br>• Poor information on the ART method used<br>• Single or twin pregnancies were not specified<br>• The difference between gender was not analyzed
Castillo-Fernandez et al., 2017	• 41 genes have different methylation<br>• Observed hypomethylation of the regulatory region of *H19* even after adjustment for smoking, maternal age, birth weight<br>• Adjusting for the ICSI method of conception, the association with these 41 DMR genes weakens considerably	• Small sample size;<br>• A role of paternal infertility cannot be excluded<br>• Results not extensible to single pregnancies<br>• The difference between gender was not analyzed
Chen et al., 2018	• Detected hypomethylation of the *KvDRM1* promoter of *CDKN1C* and hypermethylation of the *H19* DMR<br>• HTR8 cells exposed to high levels of estrogen showed hypomethylation of *KvDMR1* after 24 hours and hypermethylation of *H19* DMR after 48 hours of exposition	• Used only 6 placentas from the ART group and 5 from the SC group for the methylation study<br>• No info on the type of ART method used<br>• Not excluded impact of parental infertility<br>• All babies delivered by C-section
Chen et al., 2020	• No significant difference in global methylation and histone modifications between ART and controls<br>• Among ART subgroups, the one most similar to the control group is the IVF-ET group<br>• Analysis of DMRs and DPs shows that ART can cause epigenetic changes<br>• IVF-ET-associated DMRs could alter pathways associated with nervous, cardiovascular, and respiratory system development<br>• ICSI could impact genes involved in immune and skeletal system development<br>• Cryopreservation could impact the GTPase/Ras system involved in the genesis of preeclampsia and several genes involved in carcinogenesis	• Small sample size<br>• Effects on other tissues not evaluated
Choufani et al., 2018	• No difference in DNA methylation in the ART group compared with controls<br>• ART group and female sex were enriched with outliers<br>• The ART group outliers had methylation loss of several genes such as *GNAS*, *SGCE*, *KCNQT1OT1* and *BLCAP/NNAT*<br>• Different methylation between in vivo ART (IUI and subfertility) and in vitro ART (IVF and ICSI) subgroups	• Small sample size<br>• The effect of other procedures used in ART not analyzed

	• In the *in vitro* ART subgroup methylation alteration correlated with male infertility factor and advanced paternal age<br>• Network involved were: embryonic growth, maintenance of pregnancy, development of the nervous and reproductive systems	
Choux et al., 2017	• Greater variability of DNA methylation in the cord blood and placenta of IVF/ICSI births compared with SC<br>• Hypomethylation of *KCNQ1OT1*, *H19/IGF2*, *LINE1* and *ERVFRD1* in placenta of the ART group compared with SC<br>• Reduced methylation of *H19/IGF2* DMR in IVF group compared with ICSI<br>• No difference in expression levels of imprinted genes in placenta and cord blood<br>• Increased expression of *ERVFRD1*	• Small sample size<br>• A role of parental infertility in these changes cannot be excluded
DeBaun et al., 2003	• *LIT1* hypomethylated in 5 of the 6 patients<br>• 1 of 5 patients showed hypermethylation of *H19*<br>• 1 patient showed no methylation alteration	• Small sample size<br>• No comparison with BWS born by SC<br>• No info on parental infertility<br>• The difference between ART methods was not analyzed<br>• The difference between gender was not analyzed
El Hajj et al., 2017	• DNA methylation in children born from ICSI is no different from that of naturally conceived children<br>• 4739 CpGs differently methylated even after correction for confounding factors<br>• *ATG4C* hypermethylated in both the ICSI and IVF groups, but not in the controls<br>• Trend in hypermethylation for *SNOR1* 14-9	• Small sample size<br>• Analysis adjusted only for sperm concentration therefore it cannot be certain that the paternal infertility factor has been excluded
Estill et al., 2016	• FH group shows differences with the IUI and FZ groups, which instead show similar methylation patterns<br>• Females present more hypermethylated clusters than males<br>• In the ICSI group the imprinted genes were more often differentially methylated than in the IUI and SC group<br>• Different methylation for *H19* DMR and *IGF2* DMR2 in ART groups compared to SC but not between ART groups<br>• Hypomethylation in Metastable Epialleles (MEs) of ART compared to SC	• Small sample size<br>• Poor information about the mother<br>• Effects associated with ICSI could be attributed to parental infertility<br>• Single or twin pregnancies were not specified

Feng et al., 2011	• Similar imprinted gene expression levels between ART and controls<br>• *PEG 10* and *L3MBTL* expression was higher and *PHLDA2* expression was lower in ART than in SC<br>• Only for one case subjected to ICSI was observed loss of imprinting for *L3MBTL*<br>• No difference in methylation between ART and controls	• Small sample size<br>• Single or twin pregnancies were not specified<br>• The difference between gender was not analyzed
Ghosh et al., 2017	• Methylation levels were significantly different between the ART group and controls for both *LINE1* and CCGG<br>• Methylation difference in *LINE1* between ART and controls present in the group cultured with 20% oxygen tension<br>• Methylation difference in *LINE1* between ART and controls present in the group of patients undergo-ing fresh ET<br>• Placentas of male children had a difference in *LINE1* methylation	• Small sample size<br>• Not investigated other possible confounding factors (e.g. cul-ture medium)<br>• Not information about paternal infertility
Gomes et al., 2009	• Hypomethylation of the ART group at the level of *KvDMR1* compared to controls<br>• In dizygotic twins the methylation levels were different<br>• Excluded an influence of the underlying parental infertility and the type of method in determining these differences	• Small sample size<br>The difference between gender was not analyzed
Ji et al., 2018	• No difference between groups in terms of methylation of CpGs analyzed	• Small sample size<br>• Only good quality embryos used<br>• No information on paternal infertility<br>• SC control group missing
Jiang et al., 2022	• Reduced promoter methylation levels of the *MEG3* gene<br>• Positive correlation between *MEG3* expression levels and blood pressure in children<br>• Association between estrogen levels and expression levels of *MEG3* and ET1	• Small sample size<br>• No information about paternal infertility<br>• Not analyzed difference by gender<br>• Not analyzed the differences between the various ART tech-niques
Katari et al., 2009	• Higher average levels of methylation at CpGs in cord blood and lower levels in the placenta of the IVF group compared with controls<br>• For imprinted genes, 44 CpGs were differently methylated in cord blood and 29 in the placenta of the IVF group compared to controls	• Small sample size<br>• No information on parental infertility<br>• No correlation with ART type<br>• Single or twin pregnancies were not specified

	• Observed correlation between methylation levels and expression of these genes	• The difference between gender was not analyzed
Li et al., 2011	• No difference in average methylation levels between the groups<br>• *KvDMR1* showed a greater tendency to develop methylation aberrations	• Small sample size<br>• No info on ART type<br>• No info on maternal and paternal fertility
Lim et al., 2009	• No difference in average methylation between ART and SC-born controls<br>• Children with BWS born from ART had higher frequency of facial naevus flammeus and lower frequency of omphalocele<br>• Higher frequency of LOMs for other genes not related to 11p15.5 such as *ZAC, PEG1, SNRPN*, and *DLK1* in the post-ART group than in the ART group	• Small sample size<br>• Poor information on underlying parental infertility<br>• Not analyzed differences between ART methods<br>• Single or twin pregnancies were not specified<br>• The difference between gender was not analyzed
Litzky et al., 2017	• Difference in expression of imprinted genes only in subfertility group, while no difference in expression between IVF and control group<br>• The expression levels of *NDN, GRB19*, and *CD44* had a direct correlation with their methylation levels<br>• Correlation of expression levels of these genes with birth weight	• Small sample size especially for IVF group<br>• The control group consists of AGA children, while the other two groups were SGA and LGA<br>• Not investigated the type of subfertility
Liu et al., 2021b	• DMRs between ART-assisted and naturally conceived human offspring at the whole genome-wide level<br>• DNA methylation variations were enriched in important pathways of the immune system and nervous system	• Small sample size<br>• Lack of information on maternal and paternal infertility<br>• In cord blood samples not specified whether single or twin pregnancy<br>• The difference between gender was not analyzed
Loke et al., 2015	• Hypomethylation of *AluYa5* in IVF group compared with control<br>• Weaker evidence of hypomethylation of *LINE*<br>• Weak evidence of hypomethylation of ICR *H19/IGF2* in ICSI group vs controls<br>• Correlation between periconceptional phytic acid values and promoter methylation of H19<br>• Gestational diabetes could affect methylation of *LINE1* and smoking that of *ALuYa5* and *LINE1*	• All maternal factors were investigated by a simple questionnaire<br>• No information was given about paternal infertility<br>• Samples were stored for a long time before analysis and this might have influenced methylation<br>• Because samples were collected at 72 h after birth, the effect of postnatal epigenetic changes cannot be excluded<br>• Results not extendable to single observed pregnancies
Lou et al., 2018	• In both IVF and ICSI groups observed hypomethylation of *H19* DMR, and hypermethylation of *IGF2* DMR2 and *SNRPN* DMR	• Small sample size<br>• No SC control group

	• Only in the IVF group, high mRNA expression levels of *H19* were found	• A role of male infertility in determining hypomethylation of *H19* cannot be ruled out<br>• Single or twin pregnancies were not specified<br>• The difference between gender was not analyzed
Mani et al., 2018	• In both the IVF and SC groups, preterm infants had numerous differentially methylated CpGs<br>• Possibilities for the involvement of genes implicated in trophoblastic invasion and thus implantation<br>• Hypomethylation of the body of the gene *ADAMTS12* and hypermethylation of the promoter of *ADAMTS* 16<br>• Knockout of these genes in EVT cells associated with reduced expression of metalloproteinases 2 and 9 critical for trophoblastic invasion	• Small sample size<br>• Different gestational age between the groups<br>• No information about parental infertility<br>• The difference between gender was not analyzed
Manning et al., 2000	• Adequate methylation pattern in the analyzed region<br>• None of the children followed for a period ranging from 5 months to 4 years developed PWS or AS	• Small sample size<br>• No control group<br>• Mixed twin and singleton pregnancies<br>• The difference between gender was not analyzed<br>• Not analyzed fresh or frozen ET
Melamed et al., 2015	• 733 CpGs were significantly differentially methylated between ART and control group, with a trend toward hypomethylation.<br>• The regions that are more susceptible to methylation variation were those > 650 bp from the TSSs and those outside the CpG islands<br>• The ART group generally had greater interindividual variability<br>• Among the genes affected by this different methylation are *GNAS* and *HOP*	• Small sample size<br>• The difference between gender was not analyzed<br>• Results not generalizable to all ART but only to IVF;
Nelissen et al., 2013	• CTCF6 of *H19*, the isoforms of *MEST* and the promoter of *MEG 3* were hypomethylated in the ART group compared with the SC<br>• Only hypomethylation of *H19* was associated with a 1.3-fold increase in its expression levels	• Small sample size<br>• A role of paternal infertility cannot be ruled out since most ICSIs had been done by male factor<br>• Not possible to compare ART methods<br>• The difference between gender was not analyzed
Nelissen et al., 2014	• 839 up-regulated genes and 927 down-regulated genes were observed in the IVF/ICSI group<br>• *H19* and *PHLDA2* had 1.3- and 1.5-fold increased expression levels respectively<br>• Pyrosequencing found no difference in the incidence of LOI between the two groups	• Small sample size<br>• Not considered male infertility<br>• Single or twin pregnancies were not specified

		• The difference between gender was not analyzed
Novakovic et al., 2019	• No association was found between conception with ART and altered methylation either at birth or in the adult group<br>• In infants, DMPs were concentrated in the DMRs of 3 genes *CHRNE*, *PRSS16*, and *TMEM1*<br>• Different methylation of *CHRNE* was present in all groups, including IUI and GIFT<br>• DMRs in infants not confirmed in the adult	• No information on paternal infertility<br>• The effect of postnatal epigenetic changes in the adult group cannot be ruled out<br>• The cohorts of infants and adults is different<br>• No information about parental infertility<br>• Single or twin pregnancies were not specified<br>• The difference between gender was not analyzed
Oliver et al., 2012	• No difference in the methylation of the 4 imprinted genes analyzed and the satellite 2 repeats between the ART groups and the control group<br>• Culture medium showed a correlation with SNRPN methylation levels	• Small sample size<br>• No information on other ART technique (e.g. fresh vs. frozen ET)
Penova-Vaselinovic et al., 2021	• No difference in terms of methylation between ART and SC groups<br>• Within the ART group no difference in methylation based on the type of infertility or based on fresh or frozen embryo transfer<br>• Difference in methylation between ICSI and IVF with particular involvement of the neuroactive lig-and-receptor pathway	• Whole blood analyzed and therefore no specific tissue<br>• methylation difference can be evaluated<br>• Methylations in the two groups evaluated at different times and with different methods<br>• Since these are adolescents, epigenetic changes that occurred in the course of life cannot be ruled out<br>• Single or twin pregnancies were not specified<br>• The difference between gender was not analyzed
Pliushch et al., 2015	• No difference in methylation between the two groups	• Small sample size<br>• Analysis adjusted for several risk factors, but not considering possible paternal infertility<br>• No control with spontaneous births<br>• Single or twin pregnancies were not specified<br>• The difference between gender was not analyzed
Puumale et al., 2012	• No statistically significant differences between ART and SC groups<br>• Tendency to hypomethylation in both lymphocytes and buccal cells for IGF2R<br>• Tendency to hypermethylation only in buccal cells for *IGF2* DMR0	• Twin pregnancies have not been excluded<br>• Population of children aged ≥ 7 years who may have epige-netic alterations due to their living environment<br>• Unbalanced groups

Rancourt et al., 2012	• No LOI for any of the genes analyzed between the groups<br>• In OI group difference in methylation of *H19* in the placenta, *KCNQ1* in cord blood, and *SNRPN* in both cord blood and placenta compared with control<br>• In IVF group, difference in levels of *H19, MEST*, and *SNRPN* in the placenta, and *KCNQ1* in cord blood<br>• Correlation between methylation levels of H19 and its expression	• Small sample size<br>• No information on type of OI<br>• No information on underlying parental infertility<br>• No information on type of ART used<br>• No comparison between OI and IVF
Rossignol et al., 2006	• Both ART and SC-born patients have mutations at other loci<br>• Presence of other mutations does not change the clinical phenotype	• Small sample size<br>• The methylation status of the parents' gametes and thus the role of subfertility has not been evaluated<br>• Included both single or twin pregnancy
Sakian et al., 2015	• Expression of *H19* increased while that of *IGF2* reduced in placentas of children born by ICSI and those of children born by IVF compared with that of children born by SC<br>• No difference in methylation of the 3 CpGs of the ICR1 region of *H19* between ART and SC groups<br>• No correlation between the expression levels of H19 and IGF2 and the methylation levels of *ICR1* of *H19*<br>• Negative correlation between expression of *H19* and *IGF2*	• Small sample<br>• Mixed twin and singleton pregnancies<br>• The difference between gender was not analyzed Not considered parental infertility<br>• Only a small region of *H19* was analyzed<br>• No information about other ART procedures (e.g., fresh or frozen ET)
Santos et al., 2010	• Embryos that go through maturational arrest have an increased risk of methylation aberrations<br>• In blastocysts methylation and chromatin structure is conserved	• Small sample size<br>• A SC control group is missing<br>• Impairments cannot be ruled out due to underlying parental infertility rather than ART per se
Shi et al., 2014	• Aberrant methylation is observed in 8% of embryos for *H19* DMR, 16.9% of embryos for *PEG1* DMR and 10.4% of embryos for *KvDMR1*<br>• Spermatozoa used for fertilization showed no aberrations	• It is not possible to establish a certain role of ART in determining these aberrations since there is a lack of a comparison group<br>• There is no comparison with high-quality embryos<br>• There is no analysis that has analyzed the different ART methods
Song et al., 2015	• The study showed 11 differently methylated CpGs between the 3 groups<br>• 7 CpGs differed between the ART group with oocyte donation and healthy controls.	• Small sample size<br>• No difference between ART methods investigated

	• 12 of 18 CpGs that differed between the ART group with infertility and the control group also differed between the ART group with egg donation and the control group.	• Not considered the treatment that donated oocytes may undergo<br>• The difference between gender was not analyzed
Tierling et al., 2010	• Higher methylation of *MEST* in IVF-born babies than in ICSI-born and SC-born babies<br>• This hypermethylation in the IVF group was present in both maternal blood, cord blood, and amnion/chorion tissue<br>• Observed a slight inverse correlation between *GRB10* DMR methylation and birth weight and length	• Small sample size<br>• No information about parental infertility<br>• The difference between gender was not analyzed Not specified whether single or twin pregnancy
Turan et al., 2010	• Aberrant methylation of the maternal *IGF2/H19* DMR was more common in the in vitro group<br>• The overall variance was significantly greater in the in vitro group<br>• Both *IGF2* and *H19* mRNAs levels were significantly lower in placenta from the in vitro group	• Small sample size<br>• No information about paternal infertility<br>• No explored differences between ART methods<br>• Single or twin pregnancies were not specified<br>• The difference between gender was not analyzed
Vincent et al 2016	• The methylation of the DMR of *PLAGL1* was significantly higher in the IVF group of cord blood compared to controls and also compared to ICSI even after correction with risk factors<br>• The mRNA expression was also reduced in the IVF and ICSI group compared to SC even after correction for risk factors.	• Small sample size<br>• No information about paternal infertility<br>• Lack of information on ART methods (e.g. reason for which it was performed and whether fresh embryo transfer or frozen)
White et al., 2015	• 67% of embryos at day 3 and 50% of blastocysts had imprinted methylation alterations	• Small sample size<br>• Lack of SC control group
Whitelaw et al., 2014	• Methylation of *LINE1* in both groups increases with the age of the children<br>• SNRPN methylation is increased in ICSI patients and also according to the length of infertility<br>• The methylation within the imprinted genes does not change over the first 7 years	• Small sample size<br>• Effect not attributable with certainty to ICSI<br>• Other possible confounding factors not excluded
Wong et al., 2010	• No difference in methylation of the ICR1 region of *H19* between subgroups<br>• No correlation between average methylation levels of this region and birth weight	• Small sample size<br>• No information about parental infertility<br>• Only a small region of *H19* was analyzed<br>• The difference between gender was not analyzed<br>• Single or twin pregnancies were not specified
Yoshida et al., 2013	• No difference in methylation levels among groups	• No information about parental infertility<br>• No comparison with a spontaneous conception group

		• Unclear number of samples analyzed
Zhang et al., 2019	• The umbilical veins of the IVF group have a greater contractor response to AGII than the SC group<br>• Greater expression of *AGTR1* in turn associated with a reduced methylation of the gene	• Small sample size<br>• No information about parental infertility<br>• No information on type of ART used<br>• Single or twin pregnancies were not specified<br>• The difference between gender was not analyzed

Abbreviations. AGA, Adequate for Gestational Age; ART, assisted reproductive technique; AS, Angelman Syndrome; BWS, Beckwith-Wiedemann syndrome; COS, controlled ovarian stimulation; DMR, Differentially Methylated Region; ET, embryo transfer; EVT, Extravillous trophoblast; FET, frozen embryo transfer; HTR8, Human Trophoblast 8; ICSI, intracytoplasmic sperm injection; IUI, intrauterine insemination; IVF, in vitro fertilization; LGA, Large for Gestational Age; LOM, Loss of Methylation; OI, ovulation induction; ORF, open reading frame; PWS, Prader Willi syndrome; SGA, Small for Gestational Age; SC, spontaneous conception, NR, not reported.

Genes: *ADAMTS, ADAM Metallopeptidase With Thrombospondin Type 1 Motif 1; AGTR1, angiotensin II receptor type 1; ALU, Arthrobacter luteus; ARRDC4, Arrestin Domain Containing 4; ATG4C, Autophagy Related 4C Cysteine Peptidase; BLCAP, BLCAP Apoptosis Inducing Factor; CDKN1C, Cyclin-dependent kinase inhibitor 1C; CHRNE, Cholinergic Receptor Nicotinic Epsilon Subunit; DLK1, Delta Like Non-Canonical Notch Ligand 1; DLX5, Distal-Less Homeobox 5; ERVFRD1, Endogenous Retrovirus Group FRD Member 1; GNAS, guanine nucleotide-binding protein; GRB10, Growth Factor Receptor Bound Protein 10; HERV-FRD, Human Endogenous Retrovirus FRD; IGF2, insuline-like growth factor 2; KCNQ1, Potassium Voltage-Gated Channel Subfamily Q Member 1; KCNQ1OT1, KCNQ1 Opposite Strand/Antisense Transcript 1; KvDMR1, Potassium Voltage Differentially Methylated Region 1; L3MBTL, Lethal(3) Malignant Brain Tumor-Like protein; LINE1, Long Interspersed Nuclear Elements 1; LIT1, Long QT Intronic Transcript 1; MEG3, Maternally Expressed Gene 3; MEST, Mesoderm Specific Transcript; NDN, Necdin; NNAT, neuronatin; GNAS antisense; PEG1, Paternally expressed gene 1; PEG3, Paternally expressed gene 3; PEG10, Paternally expressed gene 10; PHLDA2, Pleckstrin Homology Like Domain Family A Member 2; SGCE, Sarcoglycan Epsilon; SNRPN, Small Nuclear Ribonucleoprotein Polypeptide N; ZAC, Zinc-Activated ion Channe.*
